# The MaoP/*maoS* Site-Specific System Organizes the Ori Region of the *E*. *coli* Chromosome into a Macrodomain

**DOI:** 10.1371/journal.pgen.1006309

**Published:** 2016-09-14

**Authors:** Michèle Valens, Axel Thiel, Frédéric Boccard

**Affiliations:** Institute for Integrative Biology of the Cell (I2BC), CEA, CNRS, Univ. Paris‐Sud, Université Paris‐Saclay, Gif‐sur‐Yvette, France; Universidad de Sevilla, SPAIN

## Abstract

The Ori region of bacterial genomes is segregated early in the replication cycle of bacterial chromosomes. Consequently, Ori region positioning plays a pivotal role in chromosome dynamics. The Ori region of the *E*. *coli* chromosome is organized as a macrodomain with specific properties concerning DNA mobility, segregation of loci and long distance DNA interactions. Here, by using strains with chromosome rearrangements and DNA mobility as a read-out, we have identified the MaoP/*maoS* system responsible for constraining DNA mobility in the Ori region and limiting long distance DNA interactions with other regions of the chromosome. MaoP belongs to a group of proteins conserved in the Enterobacteria that coevolved with Dam methylase including SeqA, MukBEF and MatP that are all involved in the control of chromosome conformation and segregation. Analysis of DNA rings excised from the chromosome demonstrated that the single *maoS* site is required *in cis* on the chromosome to exert its effect while MaoP can act both *in cis* and *in trans*. The position of markers in the Ori region was affected by inactivating *maoP*. However, the MaoP/*maoS* system was not sufficient for positioning the Ori region at the ¼–¾ regions of the cell. We also demonstrate that the replication and the resulting expansion of bulk DNA are localized centrally in the cell. Implications of these results for chromosome positioning and segregation in *E*. *coli* are discussed.

## Author Summary

The Ori region from bacterial chromosomes plays a pivotal role in chromosome organization and segregation as it is replicated and segregated early in cell division cycle and its positioning impacts the cellular organization of the chromosome in the cell. The *E*. *coli* chromosome is divided into four macrodomains (MD) defined as large regions in which DNA interactions occurred preferentially. Here we have identified a new system responsible for specifying properties to the Ori MD. This system is composed of two elements: a cis-acting target sequence called *maoS* and a gene of unknown function acting in *trans* called *maoP*. Remarkably, MaoP belongs to a group of proteins conserved only in Enterobacteria that coevolved with the Dam DNA methylase and that includes the MatP protein structuring the Ter macrodomain and the SeqA and MukBEF proteins involved in the control of chromosome conformation and segregation. These results reveal the presence of a dedicated set of factors required in chromosome management in enterobacteria that might compensate, at least partially, for the absence of the ParABS system involved in the condensation and/or segregation of the Ori region in most bacteria.

## Introduction

The size of genomes with respect to cellular dimensions imposes the need for extensive chromosome condensation that is compatible with the genome replication and the expression of genetic information. The bacterial chromosome is organized at multiple levels from the genome content (including the distribution of DNA motifs, genes and replication arms), to chromatin composition, supercoiling domains and large chromosomal domains [1,2]. The *E*. *coli* chromosome is divided into four large domains called macrodomains (MDs) [3,4]. MDs are defined as large regions in which DNA interactions occurred preferentially, whilst DNA interactions between different MDs are restricted. The Ori MD contains the origin of replication *oriC*, the opposite Ter MD contains the replication terminus and the chromosome dimer resolution *dif* site, whilst the Left and Right MDs flank the Ter MD. Two non-structured (NS) regions (NS^Right^ and NS^Left^) flank the Ori MD. DNA sites within the NS regions can interact with both flanking MDs [4].

MD organization influences the segregation of sister chromatids and the mobility of chromosomal DNA [5]. Each MD occupies a specific territory inside the nucleoid and is segregated with specific properties [5]. The molecular details of Ter MD organisation and segregation have previously been uncovered. The protein MatP (Macrodomain Ter protein) binds to a specific DNA sequence, termed *matS*, that is repeated 23 times within the 800 kb Ter region [6,7]. Furthermore, the interaction of MatP with the division apparatus associated protein ZapB promotes the anchoring of the Ter MD at mid-cell to control segregation of the Ter MD [8]. The mobility of DNA markers in MDs is highly reduced compared to NS regions. In the Ter MD, constraints on DNA mobility resulted both from the binding of MatP to *matS* sites and from its interaction with the divisome. The mechanisms responsible for structuring and constraining DNA mobility in other MDs have not been described.

The understanding of bacterial chromosome segregation has improved considerably in recent years [1]. The Ori region from bacterial chromosomes plays a pivotal role in chromosome organization and segregation as it is replicated and segregated early in cell division and its positioning impacts the cellular organization of the chromosome in the cell [2]. In many bacterial species, ParABS play a critical role in the condensation and segregation of the Ori region. Although present on different low copy number plasmids in *E*. *coli* (e.g. F, P1)[9], ParABS or any analogous system has not been found in the chromosome of *E*. *coli* or other enteric bacteria. In *E*. *coli*, a specific *cis*-acting site called *migS* affects bipolar positioning of *oriC* but its deletion does not cause any severe defects in chromosome partitioning [10]. Different processes have been proposed to contribute to the partitioning and segregation of the *E*. *coli* chromosome, including the radial confinement of nucleoid DNA [11], *migS*-driven separation of Ori regions [10], entropic exclusion of sister chromosomes [12], differential condensation levels of the chromosomal regions [13] and a MukB-TopoIV interaction for decatenation and segregation of newly replicated Ori DNA [14]. The final destination of the Ori region is controlled and maybe responsible for the transversal organization of the chromosome in these species [15–17]. However, the mechanisms that control the positioning of the Ori region remain undefined.

Genomic rearrangements have previously been used to unravel the principles of chromosome organization [18–25]. In many cases, the recombination between inverted recombining sites was used to promote the rearrangement either using general recombination systems or site specific recombination modules. Although this method can be applied with sites dispersed all over the genome it can unwittingly affect several parameters at the same time. For example, inversion of a segment encompassing the Right and Ori MD will not only generate hybrid MDs but will also affect the orientation of genes and sequences inside hybrid MDs and the intervening segment generating possible replication-transcription conflicts [26]. To limit potential replication-transcription conflicts resulting from genetic rearrangements, we previously developed a strategy that allows the transposition of any chromosomal segment at any position in the chromosome in any orientation. Thus one can change the position of any DNA segment whilst preserving its orientation relative to the replication process [27]. These rearrangements rely on a cut-and-paste process reminiscent of a transposition event and involve the site-specific recombination module of bacteriophage λ.

In an attempt to define the mechanism responsible for structuring the Ori MD, this current study has generated new chromosome configurations by transposing different regions of the Ori MD, Right MD or NS^Right^ region without affecting gene orientation. By analyzing the mobility of transposed segments we show that different regions of the Ori MD obtained new mobility properties when present in a different genomic context. Interestingly, a specific 17 bp sequence from the Ori region, termed *maoS*, was responsible for restricting DNA mobility in adjacent regions. A gene adjacent to *maoS*, termed *maoP* (previously known as *yifE*) was also required for constraining marker mobility in the Ori region. Inactivation of *maoP* removed constraints on DNA mobility in the Ori MD and allowed long-range interaction between Ori and Right MDs. The *maoS*/MaoP system contributed to the control of Ori region choreography, as inactivation of *maoP* affected the position of Ori markers. We also demonstrate that DNA replication and the resulting expansion of bulk DNA positions the nucleoid in the middle of the cell. Implications of these results for nucleoid positioning and chromosome conformation are discussed.

## Results

### Determinants constraining mobility of markers in the Ori MD

**Fig 1:**
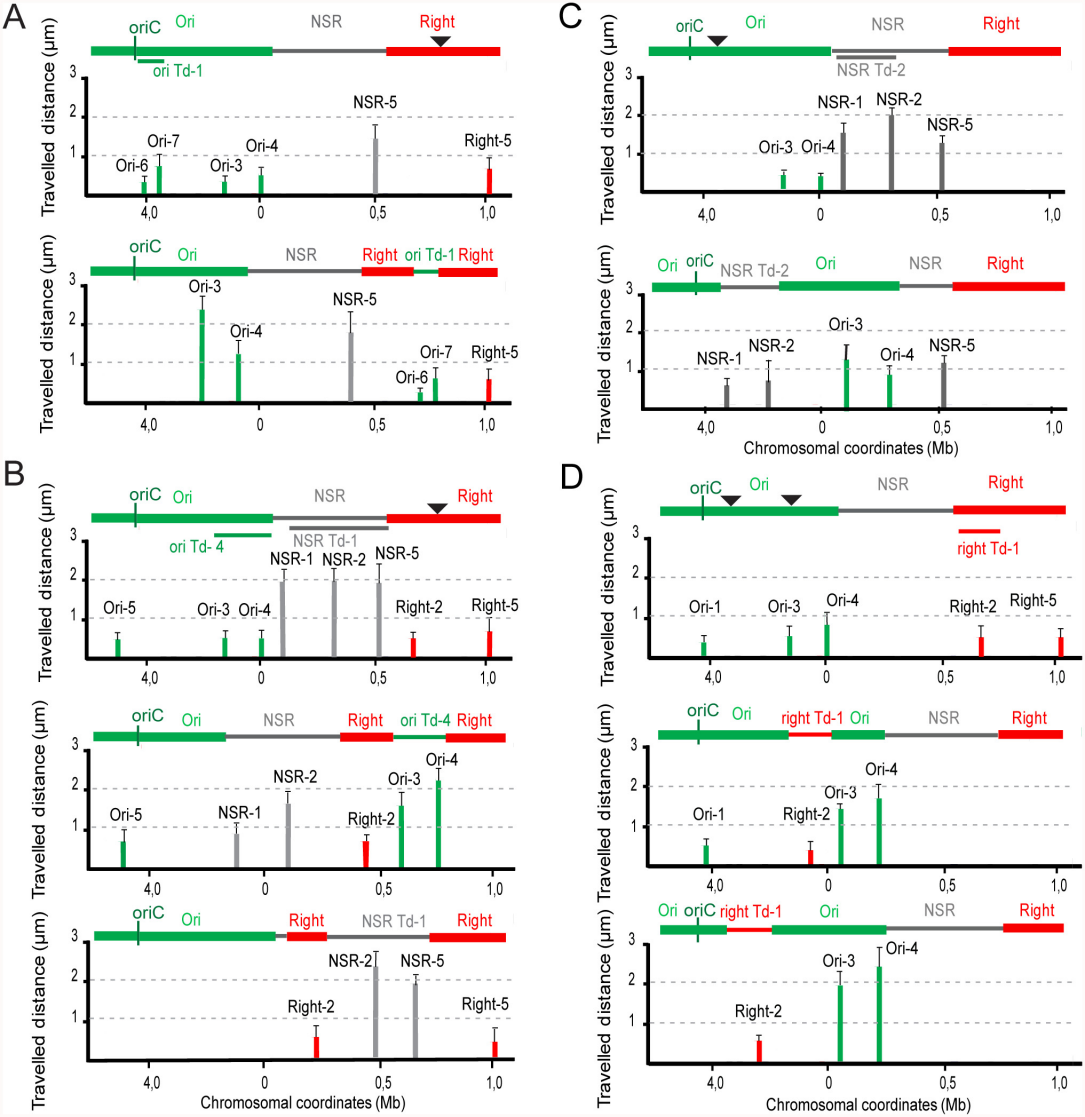
Travelled distance of different markers in strains with WT or rearranged configurations. Columns indicate the mean value with standard deviation calculated for 30 independent foci. The x axis represents the chromosome genetic map (position in Mb from *thrABC operon*). The MDs (Ori and Right) and the NS^Right^ region are indicated above the graph: the transposed segments are indicated by a line below the WT map, the insertion point by arrowhead. (A) WT strain and its derivative upon transposition of segment ori Td-1 (coordinates 3928826–4067141; strain Ori^T1^inRight^17^) in the Right MD (attB^17^). Markers Ori-6, Ori-7, Ori-3, Ori-4, NSR-5 and Right-5 are indicated. (B) WT strain and its derivatives upon transposition of the 318-kb long segment ori Td-4 (coordinates 4379216–55668; strain Ori^T4^inRight^17^) and NSR Td-1 (strain NSR^T1^inRight^17^) in the Right MD (attB^17^), respectively. Markers Ori-5, Ori-3, Ori-4, NSR-1, NSR-2, NSR-5, Right-2 and Right-5 are indicated. (C) WT strain and its derivative upon transposition of 264-kb segment NSR Td-2 (coordinates 66848 to 331520; strain NSR^T2^inOri^87.6^) in the Ori MD (87.6 min). Markers Ori-3, Ori-4, NSR-1, NSR-2 and NSR-5 are indicated. (D) WT strain and its derivatives upon transposition of 154-kb segment right Td-1 (coordinates 649460–806549) at two different positions, 92.7 min (position 4302204; strain Right^T1^in Ori^92.7^) and 87.6 min (position 4067141; strain Right^T1^inOri^87.7^), respectively. Markers Ori-1, Ori-3, Ori-4, Right-2 and Right-5 are indicated.

Our first goal was to understand if sequences within the Ori domain influence DNA mobility. To do this, we monitored movement of several chromosomal regions. The mobility of foci was determined by measuring the distance traveled by a focus from the home position at 10 sec intervals for a period of 5 min [5]. The motion of these regions was compared before and after chromosomal segments had been displaced by transposition. The transposed (Td) segments are labeled according to their original position with a numerical suffix, i.e. segments from the Ori region are called “ori Td-1”, “ori Td-2” and etcetera. A line below the genetic map indicates the transposed segments, and an arrowhead represents the insertion site. Different segments from the Ori and Right MDs or from the NS^Right^ region were inserted in the Right or Ori regions (Fig 1 and S1 Fig).

When two segments proximal to *oriC* (i.e the 138-kb ori Td-1 (Fig 1A) and the 302-kb ori Td-2 (S1A Fig)) were transposed to the Right MD at 17’, markers in the transposed Ori segment (Ori-6 and Ori-7) showed the same constraints on DNA mobility as in the original configuration (Fig 1A and S1A Fig). In this configuration, markers in the Right MD (Right-5) and in the NS^Right^ region (NSR-5) also showed the same mobility as in the WT context. In contrast, other markers in the Ori region not involved in the transposition event (markers Ori-3 and Ori-4) displayed a higher mobility (Fig 1A). Transposition of segments distal to *oriC* (i.e the 318kb ori Td-4 (Fig 1B) and the 143kb ori Td-3 (S1 Fig)) to the same locus in the Right MD resulted in transposed Ori markers (Ori-3 and Ori-4) with an increased mobility at their new location (Fig 1B and S1B Fig). The mobility of Ori markers not involved in the transposition remained unchanged. Interestingly, the marker NSR-1 in the NS^Right^ region, that was positioned closer to *oriC*, had a reduced mobility compared to the WT context. Altogether, these data indicate that the Ori region is composed of heterogeneous parts that have altered properties in different genetic contexts. In conclusion determinants responsible for limiting DNA mobility are not scattered homogeneously through the Ori region. Instead they are present in the ori Td-1 segment and constraints on DNA mobility are spread to adjacent segments.

The results described above suggested that constraints in the Ori region can spread to NS markers if they are brought closer to *C*. To test this hypothesis, a 264kb segment from the NS^Right^ region (NSR Td-2) was transposed to two different positions (respectively 92.7 min and 87.7 min) in the Ori region (Fig 1C and S1C Fig, respectively). At both positions, the mobility of markers NSR-1 and NSR-2 in the transposed segment was constraint whilst the mobility of a marker at its original position (NSR-5) was not affected (Fig 1C and S1C Fig). The mobility of markers originally located in the Ori region (Ori-3 and Ori-4) were increased following transposition even though they did not acquire the mobility of a NSR marker located the same distance from *oriC* in the WT configuration (Fig 1C). Finally, analysis of NSR-2 and NSR-5 mobility in a configuration where a large segment from the NS^Right^ region (NSR Td-1) is transposed within the Right MD (Fig 1B) indicated that all markers displayed the same mobility as in the WT strain. In conclusion the insertion of segments from either the Ori region or the NS^Right^ region into the Right MD had no consequences on the mobility of loci of the Right MD.

### Right segments impede spreading of constraints originating from the Ori region

To determine the consequences of transposing a segment of the Right MD into the Ori region, we analyzed the mobility of various markers originally located in the Right and Ori MDs following chromosome rearrangement. A 154-kb segment (right Td-1) (Fig 1D) and a 293-kb segment (right Td-2) (S1D Fig) were transposed into two different positions (respectively 92.7 min and 87.7 min) in the Ori MD. The mobility of markers in the transposed segment from the Right MD was unchanged. Interestingly the Ori markers (Ori-3 and Ori-4) that were separated from *oriC* by the transposed Right MD segment Right Td-1 had increased mobility (Fig 1D). Locus Ori-1, whose position remained unchanged, had similar constraints on DNA mobility as in the WT context (Fig 1D). Similar results were obtained for markers Right-5, Ori-3 and Ori-4 after insertion of Right Td-2 at 92.7 and 87.6 min (S1D Fig). In conclusion, segments from the Right MD were able to impede the transmission of mobility constraints originating from the region proximal to *oriC*.

### Mapping *maoS*, the organizing region of the Ori MD

**Fig 2:**
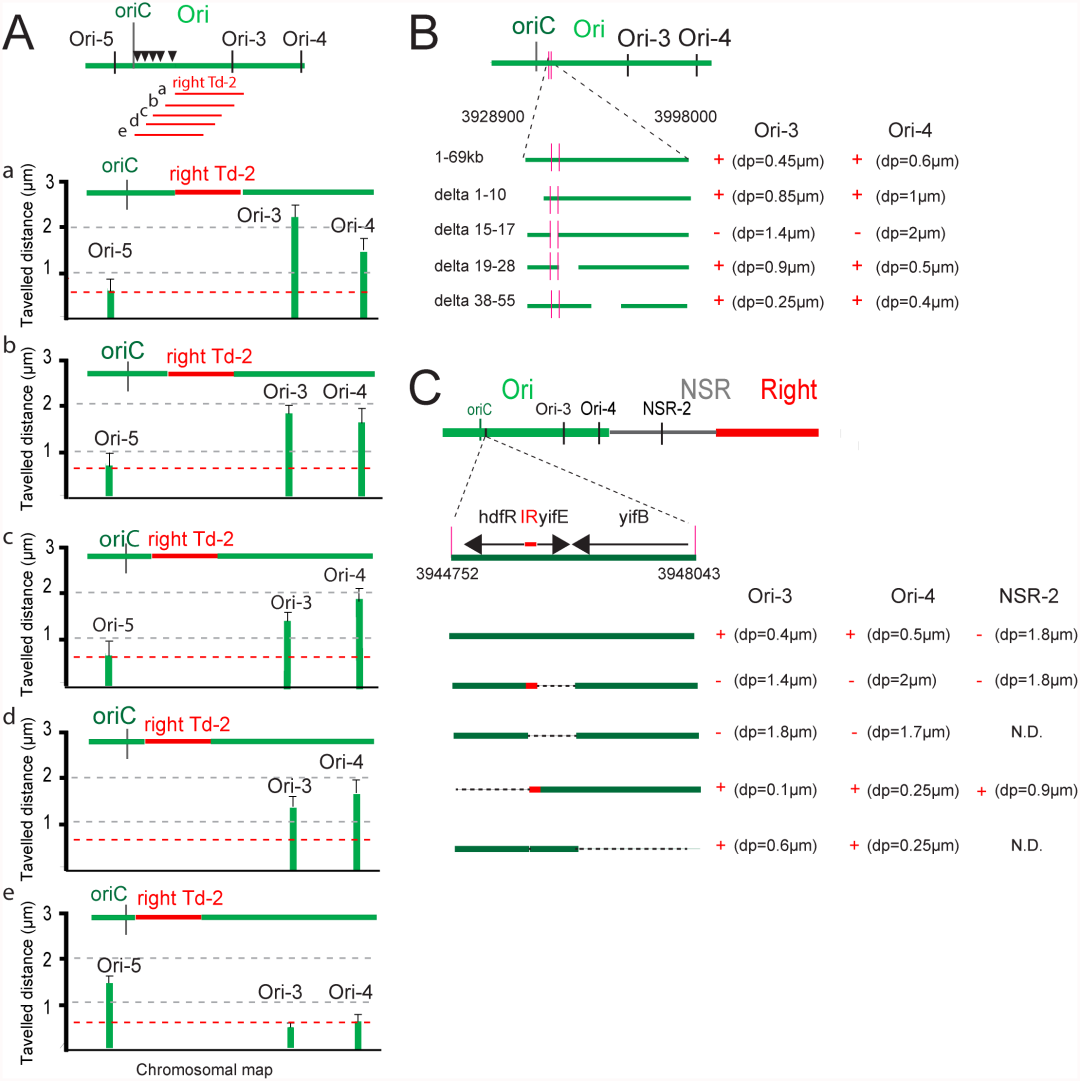
Mapping of determinants required for Ori MD structuring. (A) Travelled distance of different markers in strains with a WT or rearranged configurations carrying a segment right Td-2 inserted at various positions: 4067141 (a), 4024867 (b), 3998022 (c), 3947900 (d) and 3928826 (e) in the Ori MD. The representation is the same as in Fig 1. Markers Ori-3, Ori-4 and Ori-5 are indicated. A reference dashed red line indicates the average mobility of markers Ori-3, Ori-4 and Ori-5 in a wt context. (B) Effect of deletions spanning the 69 kb region (from coordinates 3928900–3998000) on Ori-3 and Ori-4 mobility. The extent of the region remaining upon the deletion is indicated. Deletion of the leftmost 10 kb (coordinates 3929000–3939000, from gene *rbsD* to *yieP*), of the 10 kb region between *ilvL* and *ppiC* (coordinates 3948000–3957000) and of the 16 kb region from *wzzE* to *aslB* (coordinates 3967000–3985000) had no strong effects on the mobility of markers Ori-3 and Ori-4; a strong increase in mobility of Ori-3 and Ori-4 was observed when the 2 kb region encompassing gene *hdfR*, *yifE* and *yifB* (coordinates 3944500–3946500) was deleted. Mobility of markers Ori-3 and Ori4 are indicated; the loss of constraints are indicated by “-”and DNA constraint indicated by “+”. (C) Effect of deletions spanning the *hdfR-yifE-yifB* region (from coordinates 3944752–3948043) on Ori-3, Ori-4 and NSR-2 mobility. The extent of the region remaining upon the deletion is indicated. Mobility of markers Ori-3, Ori4 and NSR-2 are indicated; the absence of constraint is indicated by “-”and DNA constraint indicated by “+”.

By utilizing the ability of transposed Right MD segments to increase the mobility of Ori MD markers, we attempted to map the determinants required for constraining DNA in the Ori MD. By inserting a segment from the Right MD at different positions in the Ori region and measuring the mobility of markers on either side of the transposed segment, it was predicted that marker mobility would be affected relative to their position to DNA constraining determinants. The 293-kb right Td-2 segment was transposed at five positions in the Ori region and marker mobility was assessed at the five positions: 87,7 min (a), 86.7 min (b), 86.1 min (c), 85.1 min (d) and 84.7 min (e) (Fig 2A). Insertion of right Td-2 at positions a, b, c, and d resulted in an increased mobility of markers distal from *oriC* (i.e. Ori-3 and Ori-4) whereas a marker close to *oriC* (i.e. Ori-5) was not affected (Fig 2A). When right Td-2 was inserted at 84.7 min (e position) the mobility of markers distal to *oriC* was not affected whilst the marker close to *oriC* had a higher mobility (Fig 2A). In conclusion, determinants responsible for constraining the Ori region were present in a region of 69-kb (coordinates 3928200 to 3998000) between positions d and e.

To precisely map the position of determinants constraining DNA mobility, four deletions corresponding to non-overlapping segments varying in size between 2 kb and 17 kb were generated in the 69 kb region (Fig 2B). Two regions could not be deleted because of the presence of essential genes. The deletion of the 2 kb segment (delta 15–17; coordinates 3944500–3946500) resulted in an increased mobility of the Ori MD markers (Ori-3 and Ori-4), whilst the three remaining deletions had no substantial effect (Fig 2B). This region contains three genes: *hdfR*, *yifE* and *yifB*. *hdfR* and *yifE* are divergent genes separated by a 118-bp long intergenic region and *yifB* is convergent with *yifE* (Fig 2C). To further define the determinants required for conferring Ori MD properties, additional deletions were performed and the mobility of markers Ori-3, Ori-4 and NSR-2 were measured (Fig 2C). These results demonstrate that both the *yifE* gene and the upstream intergenic region (IR) were required to constrain the mobility of markers Ori-3 and Ori-4. It is interesting to note that deletion of *hdfR* had an opposite effect as the mobility of all three markers was more constrained in this context. The deletion of *yifB* had no effect on marker mobility. In conclusion, *yifE* and the upstream intergenic region (IR) are required for constraining DNA mobility in the Ori MD.

### Two determinants are required for limiting mobility of markers in the Ori region

**Fig 3:**
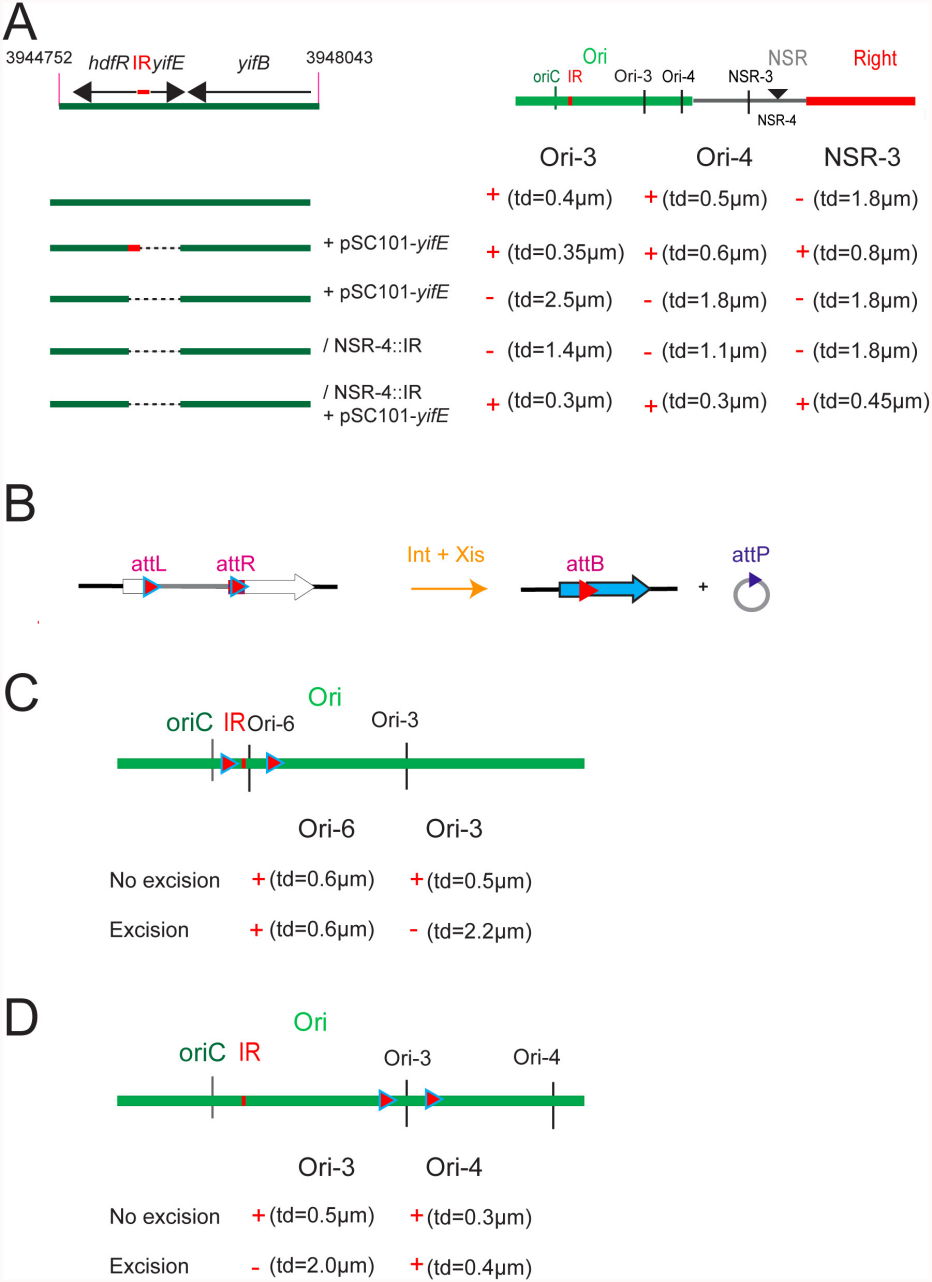
Identification of determinants required for Ori MD structuring. (A) Effect of the presence of *maoP in trans* or the insertion of *maoS* at an ectopic position on deletions spanning the *hdfR-yifE* region (from coordinates 3944752–3948043) on Ori-3, Ori-4 and NSR-2 mobility. The extent of the region remaining upon the deletion is indicated. Mobility of markers Ori-3, Ori4 and NSR-3 are indicated; the loss of DNA constraint is indicated by “-” and DNA constraint indicated by “+”. (B) Programmed excision of chromosomal segments by site-specific recombination. The two *att* sites are integrated in the chromosome in the same orientation. Excisive recombination promoted by Int+Xis results in the excision and circularization of the intervening segment carrying *att*P or *att*B (depending on the order of *att*L and *att*R sites in the chromosome). *att*L and *att*R sites are flanked by the 5’ and 3’ parts of *lacZ*, respectively. The excised segment is devoid of replication origin and is not replicated. Integrative recombination between *att*P and attB occurs at very low frequency preventing fusion of the two molecules. (C) Travelled distance of Ori markers upon excision of different segment in the Ori region. The ori Td-3 segment carries *maoS* and *maoP* indicated by a red square. Mobility of markers Ori-3, Ori4 or Ori-6 are indicated; the loss of constraints is indicated by “-”and DNA constraint indicated by “+”.

To characterize the determinants required for limiting mobility of Ori markers, constructs were generated to provide the IR and *yifE in trans* (i.e. present on a self-replicating pSC101 plasmid derivative) in different chromosomal contexts, i.e. strains deleted for *yifE* and the IR, or carrying the IR at different loci. The IR region presumably contains the promoter for *yifE*. The presence of *yifE* and the upstream IR on a replicating plasmid was not able to constrain the mobility of markers in the region (Ori-3 and Ori-4) when *yifE* and IR were deleted from the chromosome (Fig 3A). In contrast, the presence of the IR on the chromosome, either at the WT position or at NSR-1 position, together with the replicating plasmid carrying *yifE* and IR restored the constrained mobility of Ori-3 and Ori-4 markers in a *yifE* deletion mutant (Fig 3A). Interestingly, the mobility of the NSR-2 marker was constrained when the IR was inserted in the NS region (NSR-1) in the presence of the plasmid carrying *yifE*. These results demonstrate that the IR exerts its constraint on DNA mobility both in its left and right, and must be present *in cis*, whilst *yifE* can act in *trans* or *in cis*, either when present on the plasmid or inserted in the chromosome.

To identify the determinants required *in cis*, various part of the IR were tested for their ability to constrain DNA mobility of marker NSR-2 when inserted at position NSR-1. We first identified a 30 nts segment in the middle of the IR and subsequently, a 17 nts long motif with the sequence CTAATACTCCGCGCCAT was shown to limit the mobility of marker NSR-2 (S2 Fig). This motif was termed *maoS* (Macrodomain Ori Sequence). To confirm that *maoS* may act *in cis* to constrain mobility of DNA markers, we used a site-specific recombination system to loop out various parts of the Ori region from the chromosome and analyzed the subsequent effects on marker mobility. The system relies on the λ Int and Xis recombination genes. When the site-specific recombination *attR* and *attL* sites are present in a direct orientation, the recombinase promotes the excision of the intervening segment resulting in the formation of cells carrying a chromosome split in two parts (Fig 3B). As only one of the parts carries *oriC*, the cells will not be viable if essential genes are present on the non-replicated ring. The efficiency of excision varies relative to the distance separating the two *att* sites and can be assessed by plating for viable cells as only cells that did not sustain an excision event will be viable. Reinsertion of the excised ring does not occur at a high frequency [27]. To visualize the fate of excised rings immediately following excision and to measure the mobility of markers, a *parS* tag targeted by ParB-GFP was inserted on the excisable segment. The segments ori Td-1 and ori Td-3 were excised from the chromosome and the mobility of markers either present on the chromosome or on the non-replicated ring were assessed (Fig 3C and 3D). The markers located *in cis* with *maoS* (Ori-3, Ori-4, Ori-6 in the WT context, Ori-6 upon excision of ori Td-1 and Ori-4 in the context of ori Td-3) were highly constrained (Fig 3C and 3D). In contrast, the mobility of markers not *in cis* with *maoS* (Ori-3 upon ori Td-1 excision and Ori-3 upon ori Td-3 excision) was increased following excision. Combined, these results indicate that constraints in the Ori region resulted from the presence of *maoS* exerting its effect *in cis* over large distances either in the chromosome or in excised rings. In conclusion, these results identified two elements required for constraining DNA in the Ori MD: the *hdfR-yifE* IR acting in *cis* and likely acting as a target sequence and the *yifE* gene acting in *trans* likely through the encoded product. The *yifE* gene was named *maoP* (Macrodomain Ori Protein).

### Inactivation of MaoP/*maoS* specifically affects Ori MD organization

Inactivation of MaoP/*maoS* generated a number of abnormal cells (including ~5% of elongated cells) with a defect in nucleoid segregation (S3 Fig). In minimal medium supplemented with casamino acids or in LB medium at 30°C, the growth rate is slightly affected in a *maoP* mutant and the generation time is 100 min and 55 min, respectively (compared to 85 min and 50 min in WT cells). Control of replication initiation is also affected upon MaoP inactivation (S3 Fig); in minimal media, replication initiation is delayed in *maoP* cells compared to wt cells (about 50% of cells with 4 copies of *oriC* in *maoP* cells compared to more than 2/3 of cells with 4 copies of *oriC* in wt cells).

The Ori region has two properties that define it as a MD [4,5]. First, the mobility of Ori markers is lower than those of flanking NS regions and second, interactions of loci present in the Ori and Right MDs occur at a low frequency. Large intrareplichore inversions, that intermingled Ori and Right MDs, occur rarely and results in a configuration that is detrimental for growth [25]. These properties of the Ori MD were analyzed upon inactivation of the MaoP/*maoS* system.

**Fig 4:**
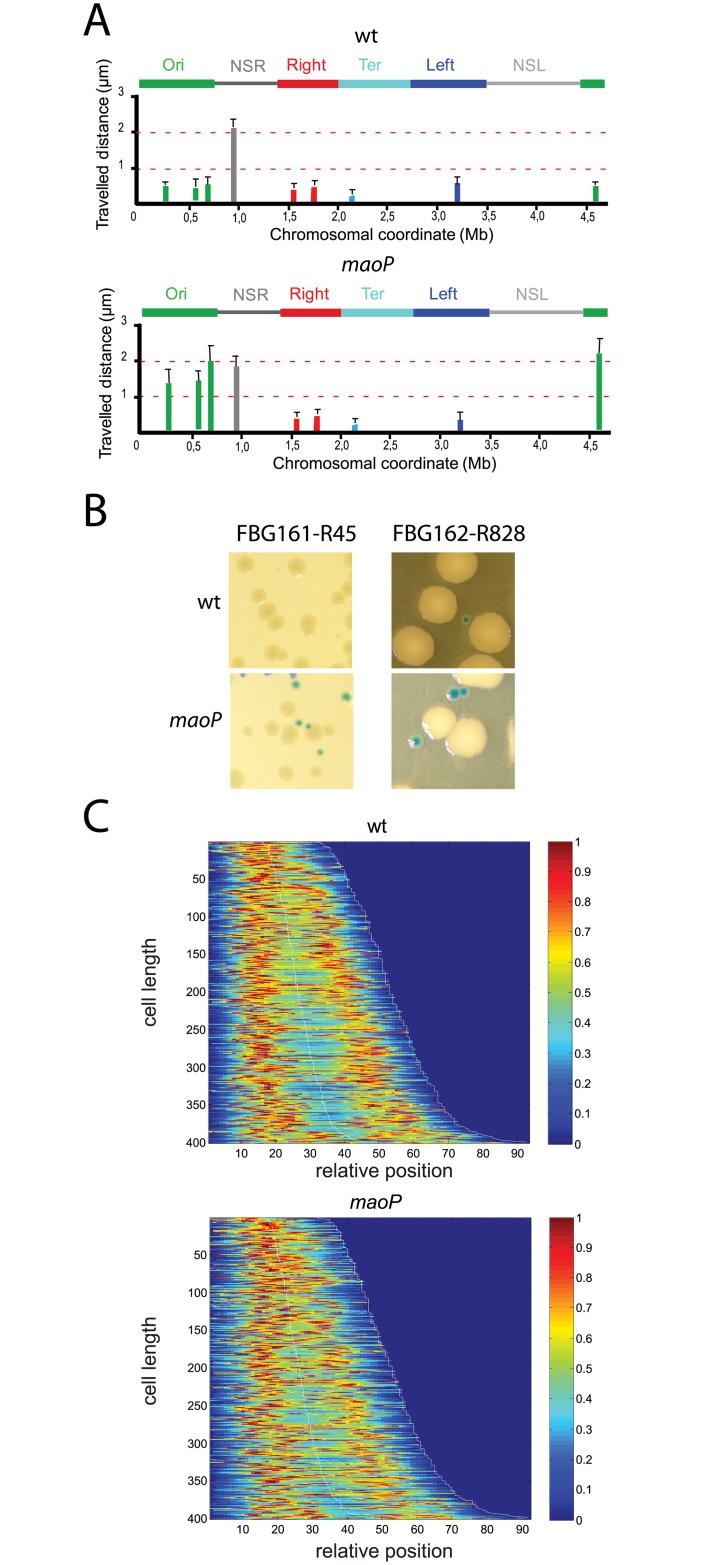
Effect of the inactivation of the MaoP-*maoS* system. (A) Travelled distance of different markers in WT strain and a *maoP* deletion derivative. Columns indicate the mean value with standard deviation calculated for 30 independent foci. The x axis represents the chromosomal genetic map (position in Mb from *thrABC operon*). The MDs (Ori, Right, Left and Ter) and the NS regions are indicated above the graph. (B) Colonies of strains carrying a normal (white colonies) or an inverted configuration (blue colonies) upon DNA inversion between Ori and Right MDs in a WT and *maoP* genetic background. (C) Positioning of chromosomal marker Ori-3 in a WT (left panel) and in a *maoP* mutant (right panel) observed in 400 cells. Cells are sorted for length, ascending from top to bottom. In the heat maps blue corresponds to low and red to high intensity. The diagram represents the relative position of the foci as a function of the cell size.

Firstly, the mobility of markers located in the Ori region were affected whilst markers in the NS region, the Right MD or the Ter MD exhibited a mobility similar to that observed in WT cells (Fig 4A). Secondly, DNA inversions between Ori and Right MDs were not detected or occurred at a very low frequency in WT cells (Fig 4B and Table 1). However, the percentage of recombinants in Ori-Right combinations was increased in *maoP* deletion mutants (Fig 4B and Table 1). This increase is specific for interactions between the Ori and Right MDs as no differences were seen for interactions between the Right and Left MDs or between the Right and Ter MDs (Table 1). Furthermore, *maoP* inactivation increased the size of the colonies with the inverted chromosomes with respect to inversions in a WT background (Fig 4B). Combined, the results demonstrate that MaoP specifies Ori MD properties including the constraint mobility of Ori markers and restrictions on long distance interactions with loci in the Right MD.

**Table 1:** Inversion rates.

Strain	Configuration	wt 36°C-20 min / 37°C 10 min	*maoP* 36°C 20 min / 37°C 10 min
**FBG151 L28**	Right-Ori	0 / 0	10.4 / 28.7
**FBG162 R128**	Right-Ori	0 / 0	15.3 / 35.4
**FBG161 R45**	Right-Ori	0 /0	17.3 / 15.8
**FBG151 L14**	Right-Right	72.8 / 79.5	29.8 /54
**FBG151 L19**	Right-Right	50 / 84.8	91.7 / 93.9
**FBG146 L160**	Right-Ter	16.2 / 24.5	5.9 / 12.5
**FBG146 L18**	Right-Ter	4 / 13.7	8.3 / 12
**FBG146 L126**	Right-Left	4.1 / 6.7	4.3 / 9.2
**FBG146 L119**	Right-Left	14.3 / 15.7	6 / 14

### Inactivation of MaoP/*maoS* affects ori positioning

To determine if MaoP/*maoS* influences chromosome choreography and Ori positioning, we analyzed the number and localization of markers as a function of cell size in WT and *maoP* deletion cells. *maoP* inactivation reduced the number of cells containing 3–4 foci and increased the number cells with two foci for markers in the Ori region (Fig 4C and S4 Fig). Newborn WT cells contain an Ori-3 marker that is rapidly duplicated and segregated to ¼ and ¾ positions of the cell. In contrast, in *maoP* cells, duplication of foci occurred later in larger cells and the average distance separating the two segregated foci was reduced in medium sized cells with positioning at the home position being less precise (Fig 4C). In contrast, inactivation of *maoP* had no effect on the number and position of markers in Right and Ter MD (S5 Fig). These results indicate that MaoP/*maoS* system affects the timing, positioning and separation of markers in the Ori MD.

Two 150-kb regions in the Ori MD contain late splitting snap loci and the late separation of newly replicated DNA in these regions resulted from an interaction with SeqA [28,29]. Inactivation of MaoP had the opposite effect as it resulted in a slight delay in the separation of loci suggesting that Ori constraining by MaoP is independent of the late *seqA*-dependent segregation of snap regions (S4 Fig).

### Control of Ori region positioning at ¼–¾

The molecular bases responsible for segregation of Ori to ¼–¾ positions following replication in *E*. *coli* are uncharacterized. The excision of DNA rings from Ori region revealed that two hours after excision, either no foci were evident in ~40% of cells or the focus was localized at one pole in ~60% of cells (S1 Text and S6 Fig). These results suggest that upon excision, DNA rings accumulated at the pole and in the absence of replication were inherited linearly in the cell population. Events leading to the formation of cells with polar foci and cells without foci were visualized using time-lapse experiments (S1 Text and S6 Fig).

**Fig 5:**
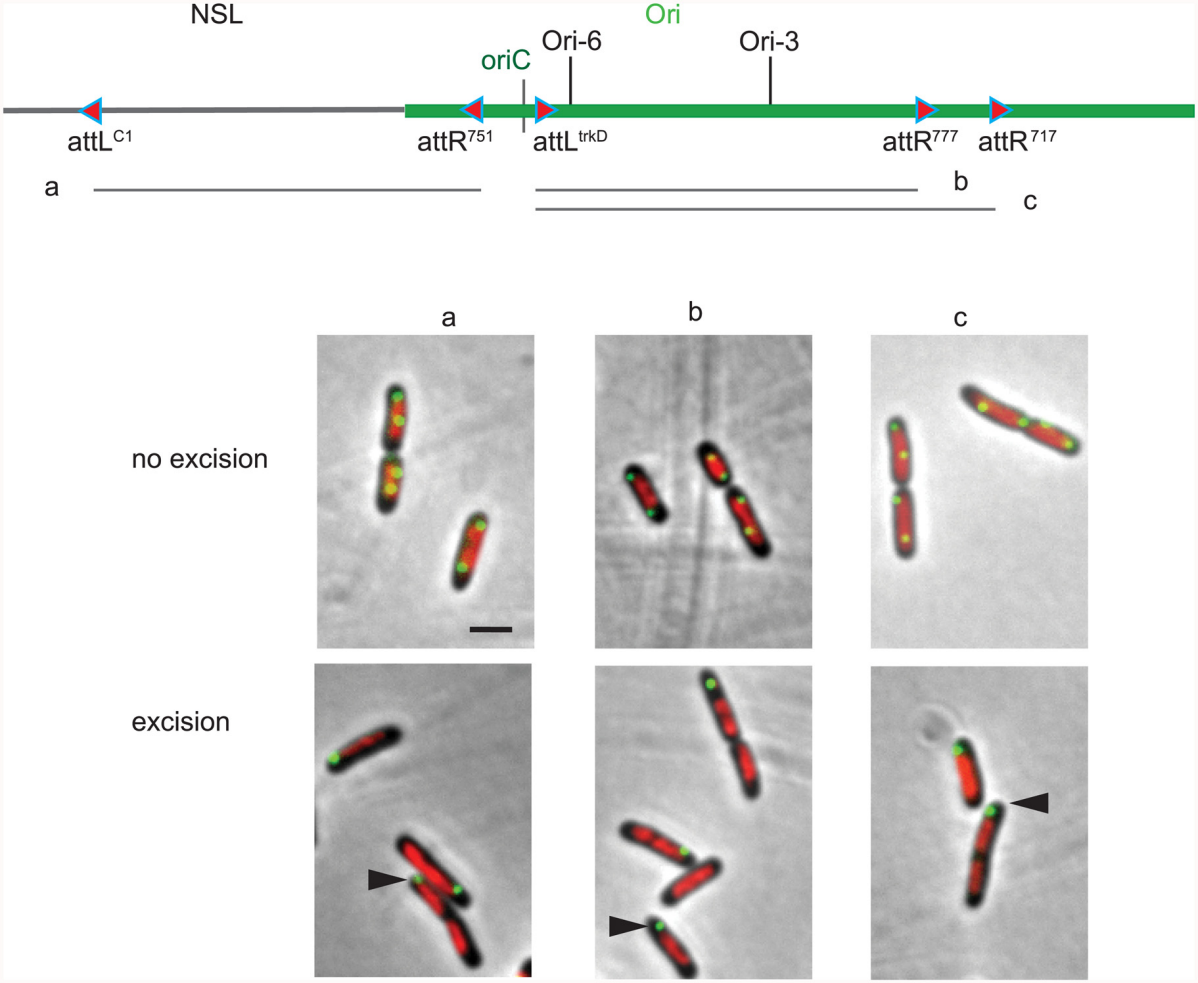
Exclusion from the nucleoid of large DNA rings looped out from the Ori region of the chromosome. Montage of merged pictures of *parS*^P1^ (green), DAPI staining (red) and phase-contrast micrographs (grey) of FBG150 cells upon excision of chromosomal DNA segments carrying the *parS* tag. The extent of the excised segment is indicated on the diagram above the micrographs (segments a, b and c). Control sample in the absence of excision is presented on the top lane (no excision). Black arrowheads indicate Ori markers present on excised rings. Scale bar indicate 2 μm.

To test whether a large segment of Ori region was required to target the Ori region at the ¼- ¾ positions, we excised large segments from the Ori region, all lacking *oriC*. In each case, a *parS* tag targeted by ParB-GFP was inserted in the excisable segment carrying *oriC* and nucleoid DNA was visualized by DAPI staining. DNA rings composed of 500–600 kb segment excised from the Ori region behave similarly to DNA rings of 150 kb in that they were found located at one cell pole and did not co-localize with nucleoid (Fig 5). The same outcome was obtained with segments originating from either side of *oriC*. Combined, these results suggest that no simple determinant was identified in the *ori* region to specify its positioning at the ¼-¾ of the cell.

**Fig 6:**
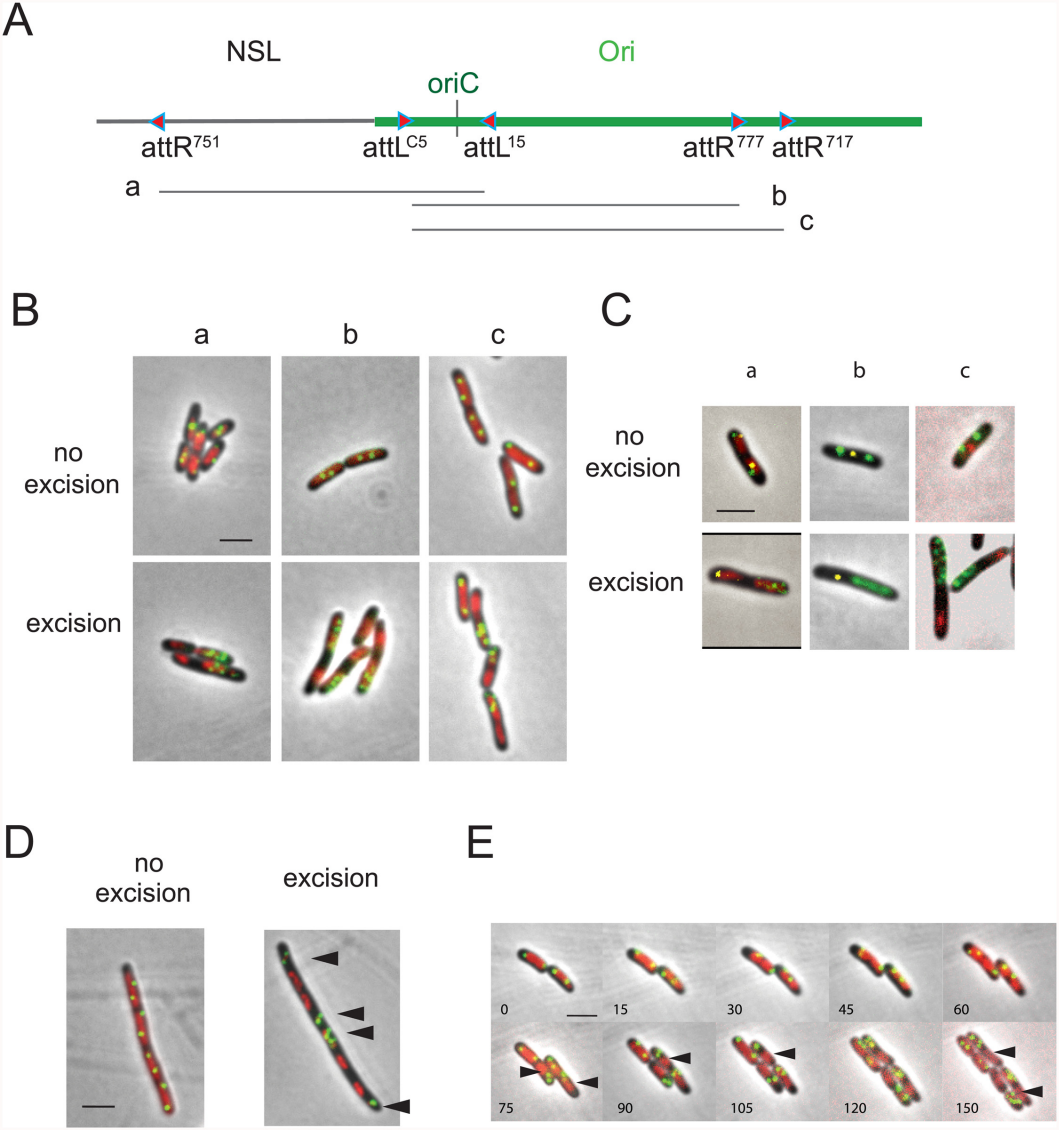
*E*. *coli* nucleoid positioning and chromosome segregation. (A) The extent of the excised segment is indicated on the diagram below the map of the NSL-Ori region (segments a, b and c). (B) Montage of merged pictures of parS^P1^ (green), DAPI staining (red) and phase-contrast micrographs (grey) of MG1655 cells upon excision of chromosomal DNA segments a, b and c carrying *oriC* and a *parS* (Ori-5 or Ori-6). Control sample in the absence of excision is presented on the top panels. (C) Montage of merged pictures of MG1655 cells upon excision of chromosomal DNA segments carrying *oriC* and the *parS* tag (Ori-5 and Ori-6; indicated in green), together with a *parS*^*T1*^ (indicated in yellow) on the remnant part either in Ori region (panel a) or in Ter region (panel b) or expressing a MatP-mCherry fusion protein (panel c), HU-mCherry staining (red; panel a) and phase-contrast micrographs (grey). Control sample in the absence of excision is presented on the top panels. (D) Montage of merged pictures of *parS*^*P1*^ Ori-6 (green), DAPI staining (red) and phase-contrast micrographs (grey) of MG1655 cells upon excision of chromosomal DNA segment b carrying *oriC* and the *parS* tag (Ori-6) in the presence of cephalexin. Control sample in the absence of excision is presented. Black arrowheads indicate excised rings carrying Ori markers. (E) Time-lapse experiment representing the dynamics of *parS*^*P1*^ Ori-6 tag upon excision of segment b. Montage of merged pictures of MG1655 cells upon excision of chromosomal DNA segments carrying *oriC* and the *parS* tag (Ori-6) and expressing HU-mCherry. Positioning of the focus was observed for 300 min with 10 min intervals. Black arrowheads indicate chromosome remnants. Scale bar indicate 2 μm.

To further explore how Ori regions segregate at ¼–¾ positions of the cell, we excised three additional segments of the Ori region carrying *oriC*. Two extended clockwise from the region close to *oriC* into the Ori MD whereas the third one extended counterclockwise from *oriC* towards the NS^Left^ region (Fig 6A). Because the distance separating the two recombining sites is large (> 500 kb), the rate of excision did not exceed 50% as estimated by the number of viable colonies. Excision gave rise to two circles, one 4 Mb remnant chromosome that is not replicated and one 500–600 kb “mini-chromosome” that is replicated via *oriC*. Excised rings and remnant chromosomes were visualized by *parS*/ParB-GFP, whilst nucleoid DNA was visualized by either DAPI staining or mCherry-HupA that uniformly labels cellular DNA.

Two hours after the excision, two types of cells were detected. Firstly, a number of cells displayed a pattern of *parS* tags and nucleoids similar to cells in which excision was not induced (Fig 6B, panel “no excision”). The number varied from experiment to experiment and presumably corresponded to cells that did not sustain an excision event. Secondly, cells contained two well separated masses of DNA, with multiple *parS* foci specifically localized with one DNA mass while the other mass was devoid of any *parS* focus (Fig 6B, panel “excision”). The presence of multiple *parS* tags indicates that 600-kb rings carrying *oriC* were able to replicate. These results suggest that, at this time of observation, multiple replicated rings remained as a single nucleoid that was separated from the 4 Mb remnant chromosome found in the other half of the cell or in the adjacent new born cell. The use of strains carrying a *parS* tag in the mini-chromosome and either another FROS tag on the remnant chromosome (either in Ori region or in Ter region) or carrying a MatP-mCherry fusion protein confirmed the separation of mini-chromosomes carrying *oriC* from the remnant chromosome (Fig 6C). The position of Ori markers suggested that rings carrying *oriC* separated at the edge of the nucleoid. The separation of circles carrying *oriC* from the 4-Mb non-replicating DNA was visualized by excising DNA segments in the presence of cephalexin to block cell division. Excised rings carrying Ori markers (indicated by arrowheads) were clearly separated from the 4-Mb non-replicating rings (Fig 6D).

Movies recapitulating the steps leading to the above situation were recorded using HU-labeled nucleoid and markers located in the excised rings carrying *oriC* (Fig 6E). Images were taken every 15 minutes during a 4h period after recombinase induction. Seventy five minutes after excision, *parS* foci were separated from nucleoid DNA that was devoid of Ori markers (indicated by red arrowheads) (Fig 6E). At subsequent times (105, 120 and 150 min), the number of fluorescent foci as well as the amount of DNA localized with Ori markers increased. At times 120 min and 150 min, small nucleoid became apparent around Ori markers (black arrowheads). Combined, these results suggest that the replication process and the expansion of nucleoid played a major role in chromosome positioning and segregation in the cell.

## Discussion

### Ori structuring

This current study presents the first description of a single *cis* acting site, *maoS*, and a *trans* acting protein, MaoP, involved in the organization of the Ori region in *E*. *coli*. Interestingly, MaoP belongs to a group of proteins involved in DNA metabolism and chromosome organization that coevolved with Dam methylase in Enterobacteria including MatP, MukBEF, SeqA, MetJ and other proteins of unknown function [30]. MatP constrains DNA over the 800-kb long Ter MD by binding to 23 *matS* target sites scattered throughout the Ter MD [7]. How a single *maoS* site constraints DNA over a distance of several hundred of kilobases is unknown, but may involve among other hypotheses a mechanism of tracking or loop extrusion (for review, see [31]) initiated at *maoS* to impose constraints over the large Ori MD. MaoP lacked homology to any characterized proteins, so how it structures the Ori MD remains unknown; furthermore, we cannot exclude the involvement of other components in this process(es).

The Ori MD has been identified by different approaches (FISH, long-distance genetic interactions, segregation pattern and mobility of markers) and has been shown to be preferentially targeted by retrotransposition of Ll.LtrB group II intron in slow growth conditions [32]. Probing the retrotransposition pattern of this element in a *maoP* mutant would reveal whether this preferential targeting is dependent of Ori constraining by MaoP.

The insertion of segments originating from the Right MD impedes the effects of MaoP/*maoS* on distal segments. This could result from determinant(s) in the Right MD that block the process or the absence of determinant(s) in the Right MD that would be required for the process to be propagated further. As the insertion of segments from NS regions acquire Ori properties when inserted in the Ori MD, it is likely that segments from the Right MD contain determinants that antagonize the propagation of Ori properties. Further structure-function analysis is required to further characterize the factors and mechanisms constraining the Ori region and to uncover how segments from the Right MD might impede this process.

### Nucleoid positioning, chromosome folding and cell organization

DNA Rings of 150–200 kb excised from various chromosomal regions accumulated at the cell pole and remained localized in this cellular space for several generations, suggesting that they could not travel across the cellular territory occupied by the nucleoid. These observations are supported by the radial confinement of the nucleoid within the cell [11] and also suggest that 100 kb-long DNA polymers that are devoid of partition systems and unable to replicate do not mix with nucleoid DNA. The position of the excised ring at the time of excision was dependent on the original location in the genetic map. Rings excised from the Ori region were found preferentially at the old poles whereas rings from the Ter MD were found at midcell/new pole. Two models describing chromosome conformation and segregation have been proposed (for review [2,33]). These models rely either on the folding of the chromosome as a random coil-like polymer compacted by external crowding forces and entropy to act as the main driver for chromosome segregation [34], or on the presence of a folded chromosome, self adhering object with intrinsic structuring, interacting with various systems that control the conformation of different regions [8,11,13,35]. The presence of multiple replicating mini-chromosomes confined only in a small fraction of the cell is in favor of the second model.

In *E*. *coli*, the nucleoid DNA does not occupy the full interior cell space whilst the space at the end contains aggregated proteins [36] and is accessible to F plasmids devoid of partition system [37] or other smaller plasmids [15,38,39]. Here, large non-replicating DNA molecules accumulated in these nucleoid free territories suggesting that an unidentified system controls central positioning of the nucleoid at midcell.

### Chromosome segregation

In many bacteria, chromosome segregation relies on the highly conserved *parABS* system. In many cases, the centromere-like *parS* sequences are found near *oriC* suggesting a coordination of segregation with replication. In *B*. *subtilis*, the large Ori region including *oriC* and *parS* sequences forms a macrodomain and its 3D folding pattern plays a role in the regulation of replication initiation, chromosome organization and DNA segregation [40]. Interestingly, Enterobacteria lack *parABS* and no analogous system has been identified. The widespread conservation of *parS* across diverse bacteria suggests that Par systems evolved early in the evolution of bacterial chromosomes and were subsequently lost from the Enterobacteria. This loss may have resulted either from the acquisition of alternative segregation systems or the acquisition of new properties in DNA metabolism and chromosome management that rendered *parABS* dispensable. Here, to identify *cis*-elements required for chromosome segregation we used a strategy that, if they exist, should favor their positioning at the ¼ and ¾ positions. However, only excised segments carrying *oriC* localized to the center of the cell; furthermore, the replication process and the expansion of nucleoid played a major role in chromosome segregation and positioning. Interestingly, multiple copies of mini-chromosomes did not immediately segregate to other half of the cell or from each other. Instead they remained together in a growing bulk suggesting that DNA confinement may be important for driving chromosome segregation and/or that an interplay with cell cycle events and/or cellular structures are required for efficient chromosome segregation.

## Materials and Methods

### Strains and media

The bacterial strains and plasmids used in this study are listed in Tables 2–4 and in S1 Table. *E*. *coli* strains were grown at 30°C in Lennox broth (rich medium), or in minimal medium A supplemented with 0.12% casaminoacids and 0.2% glucose. Antibiotics were added when necessary. The different transpositions targeted in the chromosome were performed as in [27]. The deletions and the insertions of specific segments were done by the one-step technique in strain DY330 and transduced into working strains as previously described [41,42]. Constructed strains were verified by PCR. Flow cytometry analyses were performed as described before [5].

**Table 2:** Strains used in transposition experiments.

Strain name	Transposition coordinates			Configuration after transposition^a^	reference
	attL	attR	attB’		
FBG146 trkD^attL^ 719^attR^ attB^17^ (Ori^T1^inRight^17^)	3928826	4067141	806549	ori^3757-3928^- ori^4067-46^ -NS^R^- right ^651-806-^ori^3928-4067^-right^806-1206^	This work
FBG146 trkD^attL^ 828^attR^ attB^17^ (Ori^T2^inRight^17^)	3928826	4230970	806549	ori^3757-3928^- ori^4230-46^ NS^R^- right ^651-806^-ori^3928-4230^-right^806-1206^	This work
FBG146 yjeA^attL^ 42^attR^ attB^17^ (Ori^T3^inRight^17^)	4379216	4523025	806549	ori^3757-4379^- ori^4523-46^-NS^R^- right^651-806^-ori^4379-4523^-right^806-1206^	This work
FBG146 yjeA^attL^ 11^attR^ attB^17^ (Ori^T4^inRight^17^)	4379216	55668	806549	ori^3757-4379^- NSR^55-651^-right^651-806^-ori^4379-46^-NSR^46-55^-right^806-1206^	This work
FBG146 127^attR^ 14^attL^ B^O-NSR^ (NSR^T1^inRight^19^)/(Right^T1^inNS^R3.3^)	651775	914197	153000	ori- NS^R46-153^-right ^651-914^-NS^R153-651^–right^914-1206^	This work
FBG150 LC4^attL^ 756^attR^ attB^45^ (NSR^T2^inOri^92.7^)	331520	66848	4302204	ori^3757-4302^- NS^R66-331^-ori^4302-46^ NS^R46-66^-NS^R331-651^-right	This work
FBG150LC4^attL^756^attR^ attB^719^ (NSR^T2^inOri^87.7^)	331520	66848	4067141	ori^3757-4067^- NS^R66-331^-ori^4067-46^ NS^R46-66^-NS^R331-651^-right	This work
FBG150 17^attL^ 127^attR^ attB^45^ (Right^T1^inOri^92.7^)	806549	651775	4302204	ori^3757-4302^- right^651-806^-ori^4302-46^ NS^R^-right^806-1206^	This work
FBG150 17^attL^ 127^attR^ attB^719^ (Right^T1^inOri^87.7^)	806549	651775	4067141	ori^3757-4067^- right^651-806^-ori^4067-46^ NS^R^-right^806-1206^	This work
FBG150 LC13^attL^ 17^attR^ attB^45^ (Right^T2^inOri^92.7^)	1099443	806549	4302204	ori^3757-4302^- right^806-1099^-ori^4302-46^ NS^R^-right^651-806^-right^1099-1206^	This work
FBG150 LC13^attL^ 17^attR^ attB^719^ (Right^T2^inOri^87.7^)	1099443	806549	4067141	ori^3757-4067^- right^806-1099^-ori^4067-46^ NS^R^-right^651-806^- right^1099-1206^	This work
FBG150 LC13^attL^ 17^attR^ attB^15^ (Right^T2^inOri^86.7^)	1099443	806549	4024867	ori^3757-4024^- right^806-1099^-ori^4024-46^ NS^R^-right^651-806^- right^1099-1206^	This work
FBG150 LC13^attL^ 17^attR^ attB ^R3^ (Right^T2^inOri^86.1^)	1099443	806549	3998022	ori^3757-3998^- right^806-1099^-ori^3998-46^ NS^R^-right^651-806^- right^1099-1206^	This work
FBG150 LC13^attL^ 17^attR^ attB^ilvL^ (Right^T2^inOri^85.1^)	1099443	806549	3947900	ori^3757-3947^- right^806-1099^-ori^3947-46^ NS^R^-right^651-806^- right^1099-1206^	This work
FBG150LC13^attL^ 17^attR^ attB^trkD^ (Right^T2^inOri^84.7^)	1099443	806549	3928826	ori^3757-3928^- right^806-1099^-ori^3928-46^ NS^R^-right^651-806^- right^1099-1206^	This work

**Table 3:** Strains used in inversion experiments.

Strain name	Inversion coordinates		Reference
	attL	attR	
FBG161 R45	806549	4302204	[4]
FBG161 R45Δ*terΗI*	806549	4302204	[25]
FBG161 R45 Δ*maoP*	806549	4302204	[25]
FBG161 R828	806549	4230970	This work
FBG161 R828 Δ*maoP*	806549	4230970	This work
FBG151 L28	806549	4501855	[25]
FBG151 L28 Δ*maoP*	806549	4501855	This work
FBG162 R128	806549	4408955	[25]
FBG162 R128 Δ*maoP*	806549	4408955	This work
FBG151 L14	806549	914197	[25]
FBG151 L14 Δ*maoP*	806549	914197	This work
FBG151 L19	806549	682256	[25]
FBG151 L19 Δ*maoP*	806549	682256	This work
FBG146 L160	806549	1461878	[25]
FBG146 L160 Δ*maoP*	806549	1464878	This work
FBG146 L18	806549	1914670	[25]
FBG146 L18 Δ*maoP*	806549	1914670	This work
FBG146 L126	806549	2574066	[25]
FBG146 L126 Δ*maoP*	806549	2574066	This work
FBG146 L119	806549	2803856	[25]
FBG146 L119 Δ*maoP*	806549	2803856	This work

**Table 4:** Strains used in excision experiments.

Strain name	Excision coordinates		Reference
	attL	attR	
FBG150 trkD^attL^ R719	3928826	4067141	This work
FBG150 LC4 R776	331525	4638132	This work
FBG150 LC4 R52	331525	545648	This work
FBG150 L14 R127	914197	651775	This work
FBG150 LC3 R51	1379816	1554048	This work
FBG150 LC1-R751	3841019	3411732	This work
FBG150 trkD^attL^-R717	3928826	4575276	This work
FBG150 trkD^attL^-R777	3928826	4472074	This work
FBG150 *att*L^15^-R751	4024867	3411732	This work
FBG150 *att*L^C5^-R717	3857876	4575276	This work
FBG150 *att*L^C5^-R777	3857786	4472074	This work

### Inversion assays

Inversions tests were performed as described before, for 20 min at 36°C and for 10 min at 37°C [4]. To reduce variability of the assay in the *maoP* deletion background, the integrase-excisionase module was integrated in the chromosome using phage HK022-based integrative vectors [43].

### Induction of excision

The strains used for excision carried two *att*L and *att*R sites derived from the λ site-specific integration module. The two *att* sites were inserted in the same orientation. The induction of the collision was performed at 39°C for 20 minutes and the cells were subsequently incubated at 30°C for two hours. An aliquot of the cells was plated at the same time as the non-induced strain in order to estimate lethality. When the excised segments were about 200 kb long, the level of lethality, reflecting the amount of excision, was greater than 90%. When large segments of 500 kb—600 kb were involved, the level of excision was about 50–60%. To perform time lapses analyses, cells were spread immediately after induction on slides and analyzed under the microscope. For the cephalexin experiments, cephalexin (20 μg/ml) was added 10 minutes before the induction of the excision.

### Fluorescence microscopy, mobility and positioning of DNA markers

Cultures were grown in minimal A medium in the presence of glucose and casaminoacids without IPTG to maintain a minimal level expression of *gfp-parB* present on plasmid pALA2705. For the observation of nucleoids, the cells carried a mCherry fusion upstream the *hupA* gene. Cells were plated on an agarose pad in the same medium and immediately observed under the microscope. For the short time lapse experiments, movies were recorded automatically on a Leica microscope. Autofocus was performed at every time point on the phase contrast image and GFP fluorescence was recorded on the plane with the best phase contrast focus. Image analysis was performed with ImageJ software using the manual tracking plugin (http://rsb.info.nih.gov/ij/index.html). The XY co-ordinates of the two poles and of the foci were recorded manually and processed automatically with Excel (Microsoft) software. The travelled distance (given in μm) was estimated over a period of 5 minutes by adding up the absolute values of the distances for all 10 sec interval as described before **[**5**]**. The (x,y) coordinates of the foci at every time point were recorded and the distance travelled in the 10 s interval calculated. For the mobility measurements at the home position, 30 foci were analyzed (15 cells with two foci for markers). To measure the mobility of markers, the distance travelled by various foci was recorded when markers were at home position. In the growth conditions used, at home position, markers of Ori, Right MDs as well as markers from NS regions were segregated in the two halves of the cells.

## Supporting Information

S1 Fig Travelled distance of different markers in strains with WT or rearranged configurations. Columns indicate the mean value with standard deviation calculated for 30 independent foci. The x axis represents the chromosome genetic map (position in Mb from *thrABC operon*). The MDs (Ori and Right) and the NS^Right^ region are indicated above the graph: the transposed segments are indicated by a line below the map, the insertion points by arrowheads. (A) WT strain and its derivatives upon transposition of segments ori Td-1 (strain Ori^T1^inRight^17^) and ori Td-2 (strain Ori^T2^inRight^17^) in the Right MD (attB^17^), respectively (markers Ori-6, Ori-7, Ori-3, Ori-4, NSR-5 and Right-5 are indicated). (B) WT strain and its derivatives upon transposition of segments Ori Td-3 (strain Ori^T3^inRight^17^), ori Td-4 (strain Ori^T4^inRight^17^) and NSR Td-1 (strain NSR^T1^inRight^19^) in the Right MD (*att*B^17^ and *att*B^19^), respectively (markers Ori-5, Ori-3, Ori-4, NSR-1, NSR-2, NSR-5, Right-2 and Right-5 are indicated). (C) WT strain and its derivatives upon transposition of segments NSR Td-2 in the Ori MD, at attB^92.7^ (strain NSR^T2^inOri^92.7^) and attB^87.7^ (strain NSR^T2^inOri^87.7^), respectively (markers Ori-3, Ori-4, NSR-1, NSR-2 and NSR-5 are indicated). (D) wt strain and its derivatives upon transposition of segments right Td-1 and right Td-2 in the Ori MD, at attB^92.7^ (strain Right^T1^in Ori^92.7^and Right^T2^inOri^92.7^, respectively) and attB^87.7^ (strain Right^T1^inOri^87.7^ and strain Right^T2^inOri^87.7^, respectively) (markers Ori-1, Ori-3, Ori-4, Right-2 and Right-5 are indicated).
(EPS)

S2 Fig Nucleotide sequence of the intergenic region between *hdfR* and *yifE*. (A) Various segments were tested for their ability to constrain DNA mobility of marker NSR-2 when inserted at position NSR-1 as indicated in Fig 3. Black and white bars represent segments that do and do not constrain mobility of marker NSR-2, respectively. The start codons of *hdfR* (CAC (GTG on the opposite strand)) and of *yifE* (ATG) are indicated in bold. (B) Alignment showing conserved residues of the *hdfR-yifE* intergenic region of eight enterobacteria compared to that of *E*. *coli* K12. The eight enterobacteria used are indicated: *Shigella flexneri* 2a str. 2457T; *Citrobacter rendotium* ICC168; *Salmonella enterica* subsp. *enterica* serovar Typhimurium strain SL1344RX; *Klebsiella pneumoniae* strain TH1; *Pectobacterium atrosepticum* strain JG10-08; *Serratia proteamaculans* 568; *Yersinia pestis* CO92; *Photorhabdus luminescens* subsp. *laumondii* strain HP88. Stars indicate residues strictly conserved. The 17 nt sequence *maoS* is indicated in red.
(EPS)

S3 Fig Comparison of WT and *maoP* strains (A) Comparison of microscopic analyses of WT and *maoP* strains. Coloured horizontal bars indicate the percentage of the different types of cells and nucleoids in WT (left panel) and *maoP* (right panel) strains. Green: cells containing 1 and 2 nucleoids; yellow: cells containing 4 nucleoids; red: cells with un-segregated nucleoid. (B) Control of the initiation of the replication is slightly affected in a *maoP* strain. Flow cytometry analysis of the DNA content versus the cell size of wt (top panel) and *maoP* (bottom panel) cells. Flow cytometry analysis of the DNA content versus the cell size of a wt and a *matP* strain grown until OD_600_ = 0.2 (yellow line) and grown until stationary phase (red line) in minimal medium. Rifampicin-cephalexin run out experiment of the wt and *maoP* strains grown until an O.D._600_ = 0.2 in minimal medium.
(EPS)

S4 Fig Distribution of cells carrying Ori-3 foci in WT, *maoP*, *seqA* and *maoP seqA* strains. In each panel, the distribution of cells with 1 to 5–6 foci is indicated according to cell size.
(EPS)

S5 Fig Positioning of chromosomal markers NSR-1, Right-5 and Ter-6 in a WT (left panel) and in a *maoP* mutant (right panel) observed in 400 cells. Cells are sorted for length, ascending from top to bottom. In the heat maps, blue corresponds to low and red to high intensity. The diagram represents the position of the foci (x axis) as a function of the cell length (y axis).
(EPS)

S6 Fig Exclusion from the nucleoid of DNA rings looped out of the chromosome. (A) Montage of merged pictures of *parS*^*P1*^ (green) and phase-contrast micrographs (grey) of FBG150 cells upon excision of chromosomal DNA segments carrying the *parS* tag. Excised segments correspond to parts of the Ori (segment ori Td-1), NS^Right^ (segment NSR Td-3), Right (segment right Td-1) and Ter (segment ter Td-1) regions. Control samples in the absence of excision are presented in “no excision” panels. Travelled distance of markers in their chromosomal contexts and upon excision of DNA segments (markers Ori-3, NSR-2, Right-2 and Ter-3) are indicated below the picture. Distribution of foci in WT cells or in cells that sustained excision is indicated on the Right. (B) Montage of merged pictures of *parS*^*P1*^ (green) and phase-contrast micrographs (grey) of FBG150 cells grown in the presence of cephalexin upon excision of chromosomal DNA segments carrying the *parS* tag. Excised segments correspond to parts of the Ori (segment ori Td-1) and Ter (segment ter Td-1) regions. Control samples in the absence of excision are presented on the Left. (C) Merged pictures of a time-lapse experiment showing representative cells for the segregation of the marker NSR-**5** carried by excised NSR Td-3 segment. Pictures were taken at different times after recombinase induction. Positioning of the focus was observed for 350 min with 10 min intervals. Scale bar indicate 2 μm.
(EPS)

S1 Text Supporting text. (DOCX)

S1 Table Strains and plasmids. (DOCX)

S2 Table *parS* tags used in this study. (DOCX)
