# Supplementary material for: The MaoP/maoS Site-Specific System Organizes the Ori Region of the E. coli Chromosome into a Macrodomain
Source: PLoS Genet. 2016 Sep 14;12(9):e1006309. doi: 10.1371/journal.pgen.1006309 (PMC5023128; doi:10.1371/journal.pgen.1006309)
Supplement: S1 Text — (DOCX) [file pgen.1006309.s007.docx]

**S1 Text** Exclusion from the nucleoid of DNA rings looped out of the chromosome. To further explore these events, we first excised segments from the NS^Right^, Right MD or Ter MD (S6 Fig). The number, the mobility and the positioning of markers located in the excised ring were monitored. Two hours after excision, two major types of cells were observed in all cases, being cells with no foci or cells containing a focus localized at the cell pole. A small number of cells with one or two foci co-localizing with the nucleoid likely originated from the absence of excision as their proportion is similar to the estimated viable cells. The mobility of markers present on excised rings was similar to that found in the WT chromosomal context (S6 Fig).

The proportion of cells with no focus or with one polar focus may be due to the copy number of the excised segment and/or the position of the segment at the time of excision. This was investigated by excising DNA segments from the Ori and Ter regions in cells that were incubated in the presence of cephalexin to block cell division (S6B Fig). The position of the marker was visualized as previously described whilst nucleoid DNA was visualized by DAPI staining. For rings originating from the Ori region, more than half of the foci were located at the poles of the filamentous cells (51% of cells with a polar focus, 21% with a central focus), whilst rings originating from the Ter MD produced more than 75% of the cells with a focus at the center of the cell (77% of cells with central focus and 9% with a polar focus). Combined, these results indicate that 150-kb DNA rings excised from the chromosome were excluded from the nucleoid, or were trapped close to their original position and did not freely diffuse in the cell.

To visualize the events leading to the formation of cells with polar foci and cells without foci, time-lapse experiments were performed following the excision of the NSR Td-3 segment. The fate of the NSR-5 marker was analyzed every 5 min over a 12h period following excision (S6C Fig). The plating of the cells did not give rise to colonies indicating that the excised segment carried essential genes and excision occurred at a high efficiency. 75 minutes after excision the foci were found at the cell poles with one of the two daughter cells already lacking the excised ring (S6C Fig). Upon subsequent cell divisions, polar foci were visible in only two cells and they remained at this polar position. Cells devoid of foci continued to divide for a limited number of times (6 times upon excision of the NSR Td-3 segment) before stopping growth.
