# Supplementary material for: The MaoP/maoS Site-Specific System Organizes the Ori Region of the E. coli Chromosome into a Macrodomain
Source: PLoS Genet. 2016 Sep 14;12(9):e1006309. doi: 10.1371/journal.pgen.1006309 (PMC5023128; doi:10.1371/journal.pgen.1006309)
Supplement: S1 Table — (DOCX) [file pgen.1006309.s008.docx]

**S1 Table.** Strains and plasmids

| Strain name | | Genotype | Reference | |
| --- | --- | --- | --- | --- |
| FBG146 | | MG1655 Δ*lacIZ* Δ*att*B^λ^::*aadA*-*att*R^λ^**-‘***lacZ-cat* | Valens et al., 2004 | |
| FBG150 | | MG1655 Δ*lacIZ* Δ*att*B^^::*aadA* | Valens et al., 2004 | |
| FBG150 Δ1-10 | | FBG150 Δ(3929000-3939000)::frt-cat-frt | This work | |
| FBG150 Δ15-17 | | FBG150 Δ(3944000-3946000)::frt-cat-frt | This work | |
| FBG150 Δ19-28 | | FBG150 Δ(3948000-3957000)::frt-cat-frt | This work | |
| FBG150 Δ38-55 | | FBG150 Δ(3967000-3985000)::frt-cat-frt | This work | |
| FBG150 Δ*maoP* | | FBG150 Δ(3945709-3946047) | This work | |
| FBG150 Δ(*maoS-maoP)* | | FBG150 Δ(3945590-3946072) | This work | |
| FBG150 Δ*hdfR* | | FBG150 Δ(3944752-3945590) | This work | |
| FBG150 Δ(*maoS-maoP)* NSR-1::*maoS* | | FBG150 Δ(*maoS-maoP*) maoS::71279 | This work | |
| FBG150 trkD^attL^ R719 parS^P1^::Ori-7 | 3928826 4067141 | | | This work |
| FBG150 LC4 R776 parS^P1^::NSR-2 | 331525 4638132 | | | This work |
| FBG150 LC4 R52 parS^P1^:: NSR-5 | 331525 545648 | | | This work |
| FBG150 L14 R127 parS^P1^::Right-2 | 914197 651775 | | | This work |
| FBG150 LC3 R51 parS^P1^::Ter-3 | 1379816 1554048 | | | This work |
| FBG150 LC1 R751 parS^P1^:: Ori-5 | 3841019 3411732 | | | This work |
| FBG150 trkD^attL^ R717 parS^P1^:: Ori-7 | 3928826 4575276 | | | This work |
| FBG150 trkD^attL^ R777 parS^P1^:: Ori-7 | 3928826 4472074 | | | This work |
| FBG150 attL15 R751 parS^P1^:: Ori-5 | 4024867 3411732 | | | This work |
| FBG150 LC5 R717 parS^P1^:: Ori-7 | 3857876 4575276 | | | This work |
| FBG150 LC5 R777 parS^P1^::Ori-7 | 3857786 4472074 | | | This work |
| FBG150 LC5 R717 parS^P1^::Ori-7  matP-mCherry | 3857876 4575276 | | | This work |
| FBG150 LC5 R717 parS^P1^::Ori-7  parS^PMT1^ :: Ori-4 | 3857876 4575276 | | | This work |
| FBG150 LC5 R717 parS^P1^ ::Ori-7  parS^PMT1^ ::Ter-3 | 3857876 4575276 | | | This work |
|  | |  |  | |
| Plasmid name | | **Description** | **Reference** | |
| pALA2705 | | *gfp-Δ30-parB^P1^* | Li et al. 2003 | |
| pFH2973 | | CFP-Δ30ParB^P1^ / YFP-Δ30ParB^PMT1^ | Nielsen et al., 2006 | |
| pTSA29-CXI | | pTSA29 *cI^857^*-P_R_-(*xis-int*) | Valens et al., 2004 | |
| pCP20 | | pSC101^ts^-*flp* | Datsenko and Wanner, 2000 | |
| PKD46 | | *pSC101^ts^-(γ-β-exo)* | Datsenko and Wanner, 2000 | |
| pGBM2 *maoS-maoP* | | pSC101^cop^-(*maoS-maoP)* | This work | |
