# Supplementary material for: The MaoP/maoS Site-Specific System Organizes the Ori Region of the E. coli Chromosome into a Macrodomain
Source: PLoS Genet. 2016 Sep 14;12(9):e1006309. doi: 10.1371/journal.pgen.1006309 (PMC5023128; doi:10.1371/journal.pgen.1006309)
Supplement: S2 Table — (DOCX) [file pgen.1006309.s009.docx]

S2 Table : *parS* tags used in this study

|  | gene name | position (bp) |
| --- | --- | --- |
| Ori-1 | *trkD* | 3928826 |
| Ori-3 | *aidB* | 4413507 |
| Ori-4 | *mog* | 9883 |
| Ori-5 | *yibD* | 3787240 |
| Ori-6 | *ilvL* | 3947000 |
| Ori-7 | *ubiB* | 4024865 |
| NSR-1 | *araC* | 71279 |
| NSR-2 | *crl* | 258144 |
| NSR-5 | *ybbL* | 515143 |
| Right-2 | *ybfD* | 738100 |
| Right-5 | *ycdN* | 1080438 |
| Ter-3 | *ydaA* | 1395706 |
| Ter-6 | *gusC* | 1689438 |
